# Risk Factors and Outcomes of *Clostridioides difficile* Infection in Respiratory Intensive Care Unit Patients

**DOI:** 10.1111/crj.70130

**Published:** 2025-10-09

**Authors:** Tingting Hou, Yifang Huang, Jinjun Jiang, Yuanlin Song, Shujing Chen

**Affiliations:** ^1^ Department of Pulmonary and Critical Care Medicine, Zhongshan Hospital Fudan University Shanghai China; ^2^ Department of Pulmonary and Critical Care Medicine The First Affiliated Hospital of Bengbu Medical University Bengbu China; ^3^ Department of Biostatistics, Key Laboratory for Health Technology Assessment, National Commission of Health, Key Laboratory of Public Health Safety of Ministry of Education, School of Public Health Fudan University Shanghai China; ^4^ Shanghai Key Laboratory of Lung Inflammation and Injury Shanghai China; ^5^ Shanghai Respiratory Research Institute Shanghai China

**Keywords:** *Clostridioides difficile* infection, Mortality, respiratory intensive care unit, risk factors

## Abstract

**Objective:**

This retrospective study aimed to investigate the risk factors and clinical outcomes of *Clostridioides difficile* infection (CDI) in critically ill patients admitted to the respiratory intensive care unit (RICU).

**Methods:**

We enrolled adult patients who developed diarrhea during their stay in the RICU and underwent *C. difficile* toxin testing. Patients were stratified into two groups based on test results: CDI group and *Clostridioides difficile*‐negative diarrhea (CDN) group. Risk factors for CDI and clinical outcomes were compared between the two groups.

**Results:**

The incidence of CDI in RICU patients was 8.3%. Compared with the CDN group, the CDI group had significantly lower PaO_2_/FiO_2_ (P/F) ratios (median 135 vs. 189 mmHg, *p* = 0.012) and higher rates of parenteral nutrition (83.78% vs. 60.0%, *p* = 0.012), vasopressor use (62.16% vs. 40.0%, *p* = 0.029), and analgesic administration (72.97% vs. 47.14%, *p* = 0.01). Multivariate analysis indicated that male sex was a risk factor for CDI (OR, 4.07; 95% CI, 1.25–13.26; *p* = 0.02). The CDI group had a nonsignificantly higher 60‐day mortality rate (35.14% vs. 34.29%; *p* = 0.976). Survivors of CDI patients exhibited better oxygenation (175.43 vs. 102.88 mmHg; *p* = 0.004) and lower SOFA scores (6.38 vs. 9.0; *p* = 0.017). No independent risk factors for mortality were identified. CDI patients had significantly longer RICU stays (median: 32 vs. 21.5 days, *p* = 0.02).

**Conclusion:**

In this study, male sex was independently associated with an increased risk of CDI. Although CDI did not significantly affect 60‐day mortality, it was linked to prolonged RICU hospitalization.

## Graphical abstract

The CDI incidence in RICU patients was very high, and male sex might be a risk factor. CDI was not associated with the 60‐day mortality rate if diagnosed and treated early enough, but prolonged the hospital stay. The CDI group had a lower oxygenation index; higher rates of vasopressors and analgesic drugs.

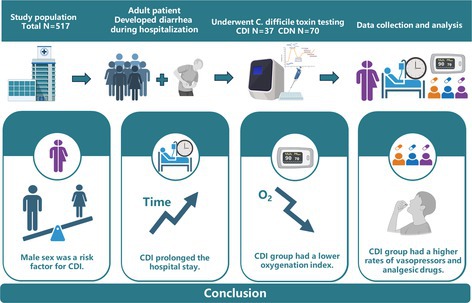

## 1 Background

*Clostridioides difficile* is a major pathogen that causes nosocomial infectious diarrhea, the incidence of which has increased significantly in recent years. *C. difficile* infection (CDI) has spread globally, imposing a serious financial burden. The main risk factors for CDI include antibiotic exposure, advanced age (over 65 years), proton pump inhibitor (PPI) treatment, and comorbidities [1, 2]. Antibiotics can reduce the diversity of intestinal microbes and disrupt the balance of the gut microbiota, leading to the proliferation of *C. difficile* [3]. The use of any antibiotic is a potential risk factor for CDI, and the incidence of CDI correlates with the duration of antibiotic exposure. Studies have shown that cephalosporins, fluoroquinolones, and clindamycin are more likely to cause CDI [4]. ICU patients are more susceptible to CDI due to severe comorbidities, broad‐spectrum antibiotics, and other invasive treatments [5]. CDI not only prolongs the hospital stay for ICU patients but also significantly increases hospitalization costs [6]. More importantly, CDI can lead to serious complications, such as electrolyte disorders, renal failure, sepsis, and even death. Therefore, the risk factors for CDI must be analyzed in ICU patients and the incidence must be reduced through effective prevention.

The respiratory intensive care unit (RICU) manages patients with severe respiratory infections, where antibiotics use is widespread. Despite the known association between antibiotics and CDI, data on CDI epidemiology in RICU populations remain limited. This study aimed to evaluate the incidence, risk factors, and mortality‐associated predictors of CDI in RICU patients.

## 2 Methods

### 2.1 Study Population and Data Collection

We conducted a retrospective cohort study involving all critically ill patients admitted to the RICU of Zhongshan Hospital (Shanghai, China) from July 2022 to April 2024. Eligible participants were older than 18 years. In this study, CDI cases met the European Society of Clinical Microbiology and Infectious Diseases (ESCMID) criteria [7], defining CDI as the presence of diarrheal symptoms alongside a positive stool test for the toxigenic *C. difficile* via polymerase chain reaction (PCR); the CDN group had *C. difficile* PCR‐negative diarrhea. Patients with diarrhea of other identified etiology were excluded. All patients with diarrhea underwent systematic *C. difficile* testing via PCR to ensure consistent case ascertainment. Once a diagnosis was confirmed, patients with nonsevere CDI received oral metronidazole or vancomycin for 14 days, whereas patients with severe CDI were treated with both drugs. Two fulminant patients also received fecal microbiota transplantation, and prevention measures such as patient isolation, terminal cleaning, and disinfection were implemented. The Clinical Research Ethics Committee of Zhongshan Hospital, Fudan University (Shanghai, China), approved this study (B2021‐031R). All procedures in this study were carried out in accordance with the rules of the ethics committee. Written informed consent was obtained from a legally authorized representative for the publication of anonymous patient information in this article.

Patient characteristics and examination results were extracted from the Hospital Information System (HIS) and included the following: demographic: age, sex, BMI, and so on; clinical parameters: comorbidities; length of hospitalization (days); 60‐day mortality rate post‐diarrhea onset; exposure history: antibiotic use within 1 month prior to diarrhea onset; medications administered within 2 weeks prior to diarrhea onset, such as glucocorticoids, PPIs, vasopressors, sedatives, analgesics, and neuromuscular blocking agents (NMBAs); and enteral nutrition and parenteral nutrition application. The Charlson comorbidity index (CCI) was used to grade comorbidities. Additionally, invasive procedures performed prior to diarrhea onset, such as mechanical ventilation, fiberoptic bronchoscopy, gastroscopy, and central venous catheterization, were documented. Laboratory results at admission, including leukocyte counts, neutrophil percentages, lymphocyte percentages, serum albumin levels, serum creatinine levels, blood urea nitrogen (BUN) levels, and C‐reactive protein (CRP) levels, were also documented. Given the respiratory focus of the RICU, the PaO_2_/FiO_2_ (P/F) ratio was included. Disease severity was quantified using the Acute Physiology and Chronic Health Evaluation II (APACHE II) score and Sequential Organ Failure Assessment (SOFA) score. Additionally, pulmonary pathogens were assessed at diarrhea onset.

## 3 Statistical Analyses

All analyses were performed with SPSS version 26 (SPSS Inc., Chicago, Illinois). Continuous variables are expressed herein as the means ± standard deviations (SDs) or as the medians and interquartile ranges (IQRs), whereas categorical variables are expressed as frequencies or percentages and were compared via the chi‐square test or Fisher's exact test. Data that were not normally distributed were analyzed via the Wilcoxon rank‐sum test, and normally distributed continuous variables were compared using the t test. The risk factors were analyzed via multivariate binary logistic regression analysis, and odds ratios (ORs) and 95% confidence intervals (95% CIs) were calculated. Only variables with a value of *p* < 0.1 in the univariate analysis were included in the final logistic regression model. Survival at 60 days after diarrhea onset was analyzed via Kaplan–Meier survival curve, and comparisons of survival rates between groups were performed by using the log‐rank test. All the statistical tests were two‐tailed, and a value of *p* < 0.05 was considered to indicate statistical significance.

## 4 Results

### 4.1 Patient Characteristics

From July 2022 to April 2024, 517 patients were admitted to the RICU of Zhongshan Hospital in Shanghai, of whom 43 (8.3%) were diagnosed with CDI, corresponding to an incidence rate of 42.85 cases per 10,000 hospital‐days. No nosocomial transmissions were detected during this period. After excluding six patients with incomplete antibiotic exposure data, we analyzed 37 CDI cases and 70 CDN cases. The CDI group had a median age of 75 years (IQR 68–80 years), and 81.08% were male, while the CDN group had a median age of 71 years (IQR 62–78.25 years), and 64.29% were male. No significant differences were observed between the two groups. All patients had been exposed to antibiotics within 30 days prior to diarrhea onset. Notably, only two CDI patients had no prior hospitalization in the 3 months before RICU admission.

### 4.2 Clinical Characteristics and Risk Factors for CDI

Univariate analysis was performed to compare clinical characteristics and laboratory test findings between two groups (Table 1). Compared with the CDN group, the CDI group exhibited a significantly lower median P/F ratio (135 vs. 189 mmHg, *p* = 0.012), potentially reflecting CDI‐associated systemic inflammation. However, the SOFA score, APACHE II score, or CCI score did not significantly differ between the two groups. CDI patients had a higher percentage of neutrophils (median: 87.96% vs. 84.76%, *p* = 0.014) and a lower percentage of lymphocytes (median: 5.1% vs. 8%, *p* = 0.008). Microbiological findings indicated a higher prevalence of *Klebsiella bacillus*, *Pseudomonas aeruginosa* , and fungi in the CDI group, whereas *Acinetobacter baumannii* , *Staphylococcus aureus* , and viruses were more common in the CDN group. However, no statistically significant differences were observed in overall pathogen distribution between the two groups (Table 2).

**TABLE 1:** Patient characteristics.

Parameter	CDI (*n* = 37) *N* (%)/median (IQR)	CDN (*n* = 70) *N* (%)/median (IQR)	Univariate analysis *p* value
Male	30 (81.08)	45 (64.29)	0.071
Age, years	75 (68.00–80.00)	71 (62.00–78.25)	0.058
Body mass index	23.44 (18.64–25.95)	21.66 (18.94–24.37)	0.336
APACHEII score on admission	20 (15.50–24.50)	18 (13.75–26.00)	0.461
SOFA score on admission	7 (5.00–10.00)	5.5 (4.00–8.00)	0.094
NRS‐2002 on admission	5 (4.00–5.50)	5 (4.00–5.00)	0.850
P/F ratio (mmHg) on admission	135 (88.30–214.00)	189 (114.58–262.35)	0.012*
CCI	4 (4.00–5.00)	5 (4.00–6.00)	0.731
Cigarette	16 (43.24)	22 (31.43)	0.225
Hospitalization in prior 3 months	37 (100.00)	68 (97.14)	0.543
Surgical intervention in prior 6 months	3 (8.11)	12 (17.14)	0.210
Antibiotic exposure in prior 1 month			
Penicillins	18 (48.65)	31 (44.29)	0.667
Cephalosporins	25 (67.57)	50 (71.43)	0.678
Carbapenems	35 (94.59)	62 (88.57)	0.504
Polypeptides	19 (51.35)	44 (62.86)	0.250
Antigungal agents	23 (62.16)	35 (50)	0.230
Aminoglycosides	4 (10.81)	9 (12.86)	1.000
Fluoroquinolones	26 (70.27)	37 (52.86)	0.082
Macrolides	2 (5.41)	5 (7.14)	1.000
Duration of antibiotic use (days)	19 (14.00–27.50)	19.5 (12.00–28.25)	0.940
Number of antibiotics received			
≥ 2	35 (94.59)	63 (90.00)	0.654
≥ 5	11 (29.73)	18 (25.71)	0.657
Device and medication use in prior 2 weeks			
PPI	33 (89.19)	67 (95.71)	0.375
Glucocorticoid	29 (78.38)	49 (70.00)	0.354
Mechanical ventilation	30 (81.08)	59 (84.29)	0.673
Central venous catheterization	34 (91.89)	64 (91.43)	1.000
Enteral nutrition	29 (78.38)	51 (72.86)	0.532
Parenteral nutrition	31 (83.78)	42 (60.00)	0.012*
Gastroscopy	2 (5.41)	5 (7.14)	1.000
Fiberoptic bronchoscopy	30 (81.08)	62 (88.57)	0.288
Vasopressors	23 (62.16)	28 (40.00)	0.029*
Sedative	28 (75.68)	57 (81.43)	0.484
Analgesic	27 (72.97)	33 (47.14)	0.01*
NMBAs	17 (45.95)	22 (31.43)	0.138
Laboratory results on admission			
Leukocyte count (×10^9^/L)	10.72 (8.24–17.60)	10.11 (7.27–14.89)	0.222
Neutrophil percentage (%)	87.96 (86.33–92.58)	84.76 (76.78–92.83)	0.014*
Lymphocyte percentage (%)	5.1 (3.20–7.60)	8 (3.75–12.13)	0.008**
Platelet (×10^9^/L)	184 (91.00–247.00)	175.5 (117.5–258.75)	0.778
D‐dimer (mg/L)	5.41 (2.07–15.80)	3.915 (1.71–8.00)	0.087
Serum albumin (g/L)	30 (29.00–34.00)	33 (28.00–35.00)	0.328
ALT(U/L)	30 (19.50–61.00)	28.5 (17.75–50.5)	0.589
AST(U/L)	31 (20.50–60.00)	35.5 (22.00–50.00)	0.724
LDH(U/L)	354 (266.50–574.25)	297 (232.00–452.75)	0.143
Bilirubin (μmol/L)	12.40 (9.10–20.85)	11.50 (7.18–18.28)	0.270
BUN (mmol/L)	9.9 (67.00–16.30)	9.75 (5.38–16.65)	0.979
Serum creatinine (μmol/L)	77 (55.50–104.50)	69 (47.50–146.25)	0.803
CRP (mg/L)	65.70 (46.10–147.10)	69.70 (28.28–142.43)	0.527
Procalcitonin (mg/L)	0.28 (0.19–1.40)	0.41 (0.19–1.46)	0.908
Hospital stay (days)	32 (18.5–41.00)	21.5 (15.00–30.50)	0.02*

*Note:* Numerical data are shown as median (interquartile range), and categorical data are described as frequency (percentage).

Abbreviations: ALT, alanine aminotransferase; APACHE II, Acute Physiology and Chronic Health Evaluation II; AST, aspartate aminotransferase; BUN, blood urea nitrogen; CCI, Charlson comorbidities index; CRP, C‐reactive protein; IQR, interquartile range; LDH, aspartate aminotransferase; NMBAs, neuromuscular blocking agents; NRS, Nutritional risk screening; PPI, proton pump inhibitor; SOFA, Sequential Organ Failure Assessment.

* *p* < 0.05.

** *p* < 0.01.

**TABLE 2:** Association of CDI and pulmonary pathogens.

Parameters	CDI (*n* = 37) *N* (%)	CDN (*n* = 70) *N* (%)	*p* value
*Klebsiella bacillus*	11 (29.73)	12 (17.14)	0.132
*Acinetobacter baumanii*	14 (37.84)	34 (48.57)	0.288
*Pseudomonas aeruginosa*	3 (8.11)	4 (5.71)	0.948
*Staphylococcus aureus*	2 (5.41)	8 (11.43)	0.504
Fungi	17 (45.95)	25 (35.71)	0.303
Virus	3 (8.11)	13 (18.57)	0.149

An analysis of the treatment strategies revealed that the CDI group had significantly higher utilization of parenteral nutrition (83.78% vs. 60%, *p* = 0.012), vasopressors (62.16% vs. 40%, *p* = 0.029), and analgesic drugs (72.97% vs. 47.14%, *p* = 0.01), compared with the CDN group. No significant differences were observed in the use of PPIs, glucocorticoids, or mechanical ventilation between the two groups. All the patients had been exposed to antibiotics, among which carbapenems, cephalosporins, and fluoroquinolones were the most commonly used. However, the amount of antibiotics used did not significantly differ between the two groups.

The CDI group had a significantly longer median hospital stay compared to the CDN group (32 vs. 21.5 days, *p* = 0.02).

To identify potential risk factors for CDI, we conducted a multivariate logistic regression analysis incorporating variables significant in univariate analysis (Table 3). The final model demonstrated that male sex was independently associated with an increased risk factor of CDI (OR: 4.07; 95% CI 1.25–13.26; *p* = 0.020).

**TABLE 3:** Multivariate analysis of variables associated with CDI.

Variables	*p* value	OR (95% CI)
Male	0.020*	4.072 (1.25, 13.264)
Age, years	0.084	1.039 (0.995, 1.086)
SOFA score on admission	0.309	0.908 (0.754, 1.093)
P/F ratio (mmHg) on admission	0.196	0.995 (0.988, 1.002)
Fluoroquinolones	0.994	0.996 (0.348, 2.849)
Parenteral nutrition	0.149	2.369 (0.735, 7.63)
Analgesic	0.682	1.278 (0.395, 4.131)
Vasopressors	0.054	2.974 (0.98, 9.029)
Neutrophil percentage (%)	0.625	0.964 (0.834, 1.116)
Lymphocytes percentage (%)	0.160	0.851 (0.679, 1.066)
D‐dimer (mg/L)	0.390	1.019 (0.977, 1.063)

Abbreviation: SOFA, Sequential Organ Failure Assessment.

* *p* < 0.05.

### 4.3 Mortality and Associated Risk Factors in CDI

**FIGURE 1:**
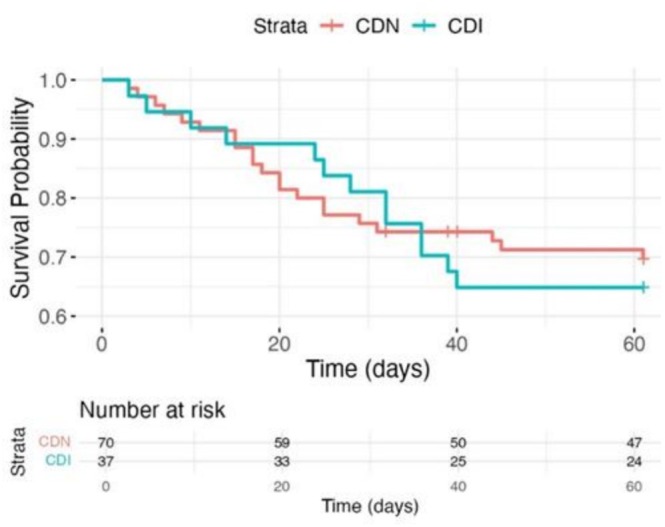
Survival curve of CDI and CDN.

We conducted a survival analysis by using the onset of diarrhea as the starting point for identifying CDI. As shown in the Kaplan–Meier survival curve, the survival rates of the two groups were basically the same in the first 2 weeks. From the third to the fifth week, the survival rate of the CDI group was higher. Considering that the treatment cycle for CDI is 2 weeks, it is speculated that some patients may have benefited from the repair of the intestinal barrier. Patients with CDI exhibited a numerically higher 60‐day mortality rate (35.14%), compared with those without CDI (34.29%), though this difference was not significant (Figure 1).

Using the American College of Gastroenterology (ACG) severity classification, we stratified CDI cases as nonsevere (72.97%, *n* = 27), severe (13.51%, *n* = 5), or fulminant (13.51%, *n* = 5). The 60‐day mortality rates increased with disease severity: 29.63% (nonsevere), 40% (severe), and 60% (fulminant); though intergroup differences were nonsignificant.

Univariate analysis identified several factors associated with higher mortality: glucocorticoid use (nonsurvivors 100% vs. survivors 66.67%, *p* = 0.032) and invasive mechanical ventilation (nonsurvivors 100% vs. survivors 70.83%, *p* = 0.038). Survivors had higher P/F ratios (175.43 vs. 102.88 mmHg; *p* = 0.004) and lower SOFA scores (6.38 vs. 9.0; *p* = 0.017). However, multivariate analysis failed to identify independent predictors of mortality (Table 4).

**TABLE 4:** Univariate and multivariate analysis of characteristics with CDI stratified for 60‐day mortality after diarrhea onset.

Parameter	Survivors (*n* = 24) *N* (%)/median (IQR)	Nonsurvivors (*n* = 13) *N* (%)/median (IQR)	Univariate analysis *p* value	Multivariate analysis *p* value
Male	18 (75.00)	12 (92.31)	0.383	
Age, years	73.29 ± 10.04	76.38 ± 9.5	0.368	
Body mass index	23.48 (17.72–27.15)	21.61 (19.92–25.22)	0.987	
APACHEII score on admission	20.33 ± 5.33	18.77 ± 5.73	0.402	
SOFA score on admission	6.38 ± 3.05	9 ± 3.06	0.017*	0.925
NRS‐2002 on admission	5 (4–5)	5 (5–5)	0.404	
P/F ratio (mmHg) on admission	175.43 ± 76.07	102.88 ± 48.18	0.004**	0.124
CCI	4 (4.5)	5 (4.6)	0.148	
Cigarette	11 (45.83)	5 (38.46)	0.739	
Device and medication use in prior 2 weeks				
PPI	21 (87.5)	12 (92.31)	1	
Glucocorticoid	16 (66.67)	13 (100)	0.032*	0.999
Mechanical ventilation	17 (70.83)	13 (100)	0.038*	0.999
Central venous catheterization	21 (87.5)	13 (100)	0.538	
Enteral nutrition	19 (79.17)	10 (76.92)	1	
Parenteral nutrition	18 (75)	13 (100)	0.072	0.999
Gastroscopy	2 (8.33)	0	0.532	
Fiberoptic bronchoscopy	17 (70.83)	13 (100)	0.038*	0.999
Vasopressors	14 (58.33)	9 (69.23)	0.724	
Sedative	16 (66.67)	12 (92.31)	0.119	
Analgesic	15 (62.50)	12 (92.31)	0.065	0.419
NMBAs	9 (37.50)	8 (61.54)	0.188	
Laboratory results on admission				
Leukocyte count (×10^9^/L)	10.37 (8.08–18.06)	11.27 (8.39–17.27)	0.718	
Neutrophil percentage (%)	88.38 ± 5.37	89.57 ± 3.98	0.487	
Lymphocyte percentage (%)	5.93 ± 3.095	5.3 ± 3.07	0.555	
Platelet (×10^9^/L)	197.5 (82.25–298.25)	142 (102–200)	0.179	
D‐dimer (mg/L)	5.32 (2.43–15.33)	6.22 (2.06–17.94)	0.863	
Serum albumin (g/L)	30 (28.25–34)	31 (29–34)	0.604	
ALT (U/L)	27 (16.50–61.00)	35 (21.50–63.00)	0.422	
AST (U/L)	32 (19.25–66.00)	29 (22–51.50)	0.913	
LDH (U/L)	321 (249–524)	398 (314.00–897.00)	0.214	
Bilirubin (μmol/L)	11.5 (7.03–19.58)	13.8 (11.00–35.10)	0.15	
BUN (mmol/L)	8.25 (4.80–15.03)	13.5 (9.35–17.80)	0.072	0.125
Serum creatinine (μmol/L)	77 (52.70–105.00)	78 (56.00–104.50)	0.838	
CRP (mg/L)	65.15 (41.83–148.25)	82.8 (52.30–157.10)	0.479	
Procalcitonin (mg/L)	0.28 (0.17–1.34)	0.42 (0.21–1.41)	0.479	
Hospital stay (days)	28.5 (17.5–41.0)	36 (21.5–42.0)	0.54	

*Note:* Numerical data are shown as median (interquartile range) or mean ± SD, and categorical data are described as frequency (percentage).

Abbreviations: ALT, alanine aminotransferase; APACHE II, Acute Physiology and Chronic Health Evaluation II; AST, aspartate aminotransferase; BUN, blood urea nitrogen; CCI, Charlson comorbidities index; CRP, C‐reactive protein; IQR, interquartile range; LDH, aspartate aminotransferase; NMBAs, neuromuscular blocking agents; NRS, nutritional risk screening; PPI, proton pump inhibitor; SOFA, Sequential Organ Failure Assessment.

* *p* < 0.05.

** *p* < 0.01.

## 5 Discussion

Our analysis demonstrated that CDI patients exhibited significantly lower P/F ratios and required vasopressor therapy more frequently compared to the CDN group. Additionally, analgesic use was markedly higher in the CDI cohort, suggesting greater disease severity and supportive care needs. No statistically significant difference was observed in 60‐day mortality between the CDI and CDN groups following diarrhea onset. However, the CDI cohort exhibited a significantly prolonged hospital length of stay compared to the CDN group.

Our retrospective study revealed a CDI incidence rate of 8.3% in the RICU, which exceeded the 0.4%–4% range reported in European ICU cohorts [8, 9], but was lower than the 10.71% incidence among Chinese ICU patients receiving enteral nutrition [10]. Notably, a multicenter study focusing on mechanically ventilated patients reported an incidence rate of 53.9 cases per 10 000 hospitalization days [11], further highlighting the variability in CDI burden across clinical settings and populations. The elevated CDI incidence in our RICU may be attributable to the high prevalence of established risk factors among enrolled patients: all received antibiotics, 83.18% (*n* = 89) underwent invasive mechanical ventilation, and 74.77% (*n* = 80) received enteral nutrition—interventions previously associated with CDI development [10, 12, 13]. Additionally, the advanced median age of our cohort (73 years, IQR: 65–79) likely contributed to this observation, given the well‐documented association between aging and CDI susceptibility.

While prior studies revealed that female are more prone to CDI [14, 15], our data paradoxically demonstrated male predominance, aligning with several ICU‐based studies [9, 10, 11, 16, 17]. This discrepancy may reflect differences in study populations (e.g., RICU vs. general hospital settings) or variations in the anatomical distribution of CDI (e.g., community‐acquired vs. nosocomial). However, the influence of antibiotic exposure on this association could not be assessed, as all participants in our cohort received antibiotic therapy, precluding comparative analysis.

Mounting evidence implicates gut microbiota dysbiosis as a central driver of CDI [18]. In critically ill patients, this dysbiosis is exacerbated by multiple iatrogenic factors: hypoxia‐induced shifts in microbial composition [19], opioid‐mediated immunosuppression and microbial alterations [20], and nutritional interventions that disrupt colonocyte metabolism and barrier function. Our findings align with this paradigm, demonstrating that CDI‐positive RICU patients exhibited higher exposure to both enteral and parenteral nutrition compared to baseline ICU rates. Notably, the preferential use of parenteral nutrition in non‐surviving CDI patients corroborates prior reports linking parenteral nutrition to increased 28‐day mortality in ICU‐acquired CDI [9], likely reflecting compounded microbiota injury and systemic metabolic dysregulation. While enteral nutrition has been established as a CDI risk factor [10], our data reveal a clinically underappreciated association between parenteral nutrition and adverse CDI outcomes. Despite mechanistic links between gut microbiota and respiratory infections via the gut‐lung axis [21], our analysis did not detect significant correlations between respiratory pathogens and CDI.

Our research findings bear resemblance to those of a prior study involving mechanically ventilated patients [22]. A systematic review and meta‐analysis carried out in the ICU demonstrated that patients with CDI experienced longer durations of ICU and overall hospital stays. Specifically, the overall hospital mortality rate stood at 32% for CDI patients, while it was 24% for non‐CDI patients. This disparity strongly suggests that CDI might be a contributing factor to the elevated mortality [23]. In another ICU‐based retrospective analysis, the 28‐day mortality was markedly higher among CDI patients compared to non‐CDI patients (27.3% vs. 9.0%). This study also indicated that the use of immunosuppressants during ICU admission was associated with an increased 28‐day mortality rate [9]. Zahar's team conducted a multi‐center prospective cohort study on 5260 ICU patients. Their results indicated that when aggressively treated, early‐stage CDI was not linked to mortality. Although the hospital stay of CDI patients was marginally extended, the difference was not statistically significant [24]. In our study, only two patients with immunodeficiency diseases (excluding HIV) were included in the CDI group. There was no significant difference in immunosuppressive therapy between the two groups during the hospitalization period. We postulate that early diagnosis and timely treatment could be the key factors accounting for the comparable mortality rates between the two groups. We classified the severity of CDI according to the ACG scores. Our results showed that the severity grading of CDI was correlated with mortality rates, which is in line with the previous research by Dionne's team [22].

Previous studies have established a link between glucocorticoid therapy and increased mortality in CDI patients, regardless of whether they were in the general ward or the ICU [9, 25]. Our study further corroborated this finding, revealing that CDI patients who received glucocorticoids had a higher mortality rate. Therefore, in clinical practice, the indications for glucocorticoid use should be strictly regulated. The results of our study showed that deceased patients had a higher SOFA score and lower oxygenation index. Another study reported that in the ICU setting, the SOFA score was an independent predictor of death in CDI patients [9]. Multiple studies have demonstrated that the oxygenation index has prognostic value for sepsis and acute respiratory failure, with a lower oxygenation index being significantly associated with increased mortality [26, 27, 28]. Collectively, the SOFA score and oxygenation index are valuable prognostic indicators for CDI patients.

As far as we know, only a limited number of articles have centered on CDI in the RICU. These studies have either reported the incidence and risk factors of CDI in the RICU or analyzed the influence of the oxygenation index on CDI. Nevertheless, we are well aware that our study comes with several limitations. Firstly, in the multivariable analyses, the covariates were carefully selected based on previous literature, clinical relevance, and univariate analyses. However, the small sample sizes could have restricted the statistical power required to identify significant associations. Moreover, this may heighten the risk of overfitting in the multivariable models. Secondly, this is a single‐center retrospective study with a relatively small sample. Consequently, the findings may not be generalizable to all healthcare institutions. The unique characteristics and patient populations of different healthcare settings might lead to different results, and our study's conclusions may not hold true across the board. Thirdly, the data were collected from patients with respiratory illnesses in the RICU. These patients often present with varying degrees of respiratory failure. Hypoxia is a common condition in such patients and can also trigger alterations in the gut microbiota. To accurately determine the specific impact of hypoxia on CDI, future research could recruit patients without hypoxia as a control group. This would help isolate the effect of hypoxia from other confounding factors. Finally, as this is a retrospective study, it is impossible to validate the causal relationship between risk factors and outcomes. Retrospective studies rely on existing data, which may lack some key information and control over variables. Therefore, multicenter, large‐sample cohort studies are urgently needed. Such studies would allow for better control of confounding factors, a more comprehensive understanding of the relationship between risk factors and CDI, and more reliable results that can be applied more widely in clinical practice.

## 6 Conclusion

Our study revealed that the incidence of CDI in the RICU is 8.3%, with male sex being identified as a risk factor for CDI. The oxygenation index value, the administration of vasopressors, treatment with analgesic medications, and the use of parenteral nutrition may be associated with CDI. The majority of CDI cases in the RICU were nonsevere. CDI was associated with a longer hospital stay but had no effect on the 60‐day mortality rate. Given the higher incidence of CDI in RICU patients, early identification and treatment are of great significance.

## Ethics Statement

All individuals or their legally authorized representatives provided informed consent to participate in this study, and approval was provided by The Clinical Research Ethics Committee of Zhongshan Hospital, Fudan University, Shanghai, China (B2021‐031R).

## Conflicts of Interest

The authors declare no conflicts of interest.

## Data Availability

The data that support the findings of this study are available from the corresponding author upon reasonable request.
